# hUC-MSCs Attenuate Acute Graft-Versus-Host Disease through Chi3l1 Repression of Th17 Differentiation

**DOI:** 10.1155/2022/1052166

**Published:** 2022-10-12

**Authors:** Weijiang Liu, Fulin Yuan, Haitao Bai, Yuanlin Liu, Xue Li, Yang Wang, Yi Zhang

**Affiliations:** ^1^Department of Experimental Hematology and Biochemistry, Beijing Institute of Radiation Medicine, Beijing 100850, China; ^2^Institute of Life Science, Hebei University, Baoding 071002, China

## Abstract

Mesenchymal stem cells (MSCs) have already demonstrated definitive evidence of their clinical benefits in acute graft-versus-host disease (aGvHD) and other inflammatory diseases. However, the comprehensive mechanism of MSCs' immunomodulation properties has not been elucidated. To reveal their potential immunosuppressive molecules, we used RNA sequencing to analyze gene expression in different tissue-derived MSCs, including human bone marrow, umbilical cord, amniotic membrane, and placenta, and found that chitinase-3-like protein 1 (Chi3l1) was highly expressed in human umbilical cord mesenchymal stem cells (hUC-MSCs). We found that hUC-MSCs treated with interferon-gamma (IFN-*γ*) and tumor necrosis factor-alpha (TNF-*α*) exhibited increased expression of Chi3l1 and concurrently repressed T-helper 17 cell (Th17) differentiation through inhibition of signal transducer and activator of transcription 3 (STAT3) activation. Furthermore, Chi3l1 knockdown hUC-MSCs exhibited impaired therapeutic efficacy in aGvHD mice with an increased inflammatory response by promoting Th17 cell differentiation, including an increase in IL-17A in the spleen, intestine, and serum. Collectively, these results reveal a new immunosuppressive molecule, Chi3l1, in hUC-MSCs in the treatment of aGvHD that regulates Th17 differentiation and inhibits STAT3 activation. These novel insights into the mechanisms of hUC-MSC immunoregulation may lead to the consistent production of hUC-MSCs with strong immunosuppressive functions and thus improved clinical utility.

## 1. Background

Mesenchymal stem cells (MSCs) are a promising treatment for regulating inflammation and inflammatory disease due to their strong immunoregulatory capacity [1]. Previous studies have identified various immunosuppressive molecules of MSCs, including indoleamine 2,3-dioxygenase (IDO) [2, 3], one of the most important candidates, along with transforming growth factor–b1 (TGF*β*) [4, 5], hepatocyte growth factor (HGF) [6], prostaglandin E2 (PGE2) [6, 7], soluble human leukocyte antigen G (HLA-G) [8], tumor necrosis factor-alpha stimulated gene 6 protein (TSG-6) [7], and exosomes [9, 10]. This knowledge regarding MSC immunobiology may explain why there are many clinical trials investigating the application of MSCs in acute graft-versus-host disease (aGvHD) and other inflammatory disease treatments [11, 12].

Continued advances in fundamental immunology, genetic engineering, gene editing, and synthetic biology have exponentially expanded the opportunities to enhance the accuracy of MSCs therapies, increase their immunomodulation potency and safety, and broaden their potential for the treatment of autoimmune diseases. For example, several studies have already demonstrated that hypoxia [13, 14], interferon-gamma (IFN-*γ*), and tumor necrosis factor-alpha (TNF-*α*) pretreatment or overexpression of immunosuppressive molecules improve MSCs' immunosuppressive capacity [15, 16]. Interestingly, a previous study also indicated that serum from aGvHD patients or interferon-gamma pretreatment MSCs significantly improves immunosuppressive activity in aGvHD [17]. There is consensus that the immunosuppressive activity does not solely rely on MSCs but may also involve the surrounding inflammatory microenvironment. In summary, the exact molecular mechanisms by which MSCs affect immune cells remain unclear. Therefore, the comprehensive immunoregulatory effects of MSCs still need to be explored.

To better understand the mechanisms underlying MSC immunomodulation capacity and to improve their clinical efficacy, we performed RNA transcriptomic profiling on MSCs isolated from human bone marrow, umbilical cord, amniotic membrane, and placenta tissue using high-throughput sequencing. Comparative analysis of the gene composition of all four MSC types revealed that human umbilical cord-derived MSCs (hUC-MSCs) exhibited abundant expression of chitinase-3-like protein 1 (Chil3l1), a secreted protein that has been implicated in several immune cell functions and inflammatory diseases [18, 19]. Gene set enrichment analysis indicated that Chi3l1 may be involved in aGvHD pathological processes and T cell proliferation and differentiation, but this observation has not been confirmed. In this study, we demonstrated that hUC-MSCs secreted Chi3l1, inhibiting T cell proliferation, in line with recent study findings [20]. In addition, we revealed that hUC-MSC-secreted Chi3l1 plays an important role in restraining Th17 cell differentiation by suppressing STAT3 activation. Importantly, an in vivo study demonstrated that Chi3l1 knockdown hUC-MSCs exhibited impaired the therapeutic activity of aGvHD, including increased clinical scores, reduced survival rate, and exacerbated tissue injury. In addition, IL-17A levels were increased in Chi3l1 knockdown hUC-MSC infusion mouse serum, spleen, and intestine. Taken together, these findings revealed that Chi3l1 is a novel immunosuppressive molecule of hUC-MSCs that regulates Th17 differentiation and is important for attenuating aGvHD symptoms.

## 2. Methods

### 2.1. Cell Preparation

hUC-MSCs were isolated from human umbilical cord as previously described [21]. Human umbilical cord (hUC) specimens were obtained from normal full-term pregnancies according to the regulations of the Research Ethics Committee of Jishuitan Hospital (Beijing, China). Residual blood, veins, and arterial vasculature were removed under sterile conditions. Umbilical cord tissue was shredded into small pieces and digested in 0.1% type II collagenase at 37°C for 45 min. The digested tissue was transferred into *α*-MEM supplemented with 10% FBS, 2 mM glutamine, 100 U/ml penicillin, and 100 mg/ml streptomycin. Fresh culture medium was added to the cell every 2 to 3 days until the adherent cells reached a confluence of approximately 80% at which point they were collected using 0.125% trypsin for subsequent expansion and characterization.

### 2.2. hUC-MSC Differentiation Assay

Adipogenic and osteogenic differentiation capacity was assessed as previously described [22]. To determine adipogenic differentiation capacity, cells (8 × 10^4^) were cultured with *α*-MEM containing 10% FBS, 10^−3^ mM dexamethasone, 0.5 mM isobutyl methylxanthine, 0.2 mM indomethacin, and 10 *μ*g/ml insulin (Sigma) for 2 weeks. An Oil red O (Sigma) staining assay was used to identify the production of adipocytes.

Osteogenic differentiation capacity was assessed by incubating cells (7 × 10^3^) with *α*-MEM containing 10% FBS with 10^−7^ mM dexamethasone, 0.5 mM ascorbic acid, and 10 mM *β*-glycerol phosphate (Sigma) for 3 weeks. Osteoblasts were identified by the presence of calcium deposits using 5% Alizarin red S (*v*/*v*) (Sigma).

### 2.3. Gene Knockdown Using shRNA

Chi3l1 expression was knocked down in human MSCs using Chi3l1-targeting shRNA carried on a lentiviral vector (GV493/hU6-MCS-CBh-gcGFP-IRES-puromycin) (GeneChem, Shanghai). The shRNA target sequence for Chi3l1 was CHI3L1-RNAi (75488-1): 5′-ACCCACATCATCTACAGCTTT-3′; CHI3L1-RNAi (75489-1): 5′-CAGCAGCTATGACATTGCCAA-3′; and CHI3L1-RNAi (75490-1): 5′-AGGTGCAGTACCTGAAGGACA-3′. hUC-MSCs were incubated with lentivirus and HitransG P (Gene Chem, Shanghai) for 8 h. Puromycin (1 mM) was added to the culture medium to select transduced cells.

### 2.4. Flow Cytometry

Cultured cell immunophenotypic analysis was performed by flow cytometry. Cells were stained with fluorophore-conjugated monoclonal antibodies according to established protocols. Briefly, PE-anti-human CD73 monoclonal antibody (Invitrogen, 12-0739-42), PE-anti-human CD105 monoclonal antibody (Invitrogen, 12-1057-42), PE-anti-human CD90 monoclonal antibody (Invitrogen, 12-0909-42), APC-anti-human CD34 monoclonal antibody (Invitrogen, 17-0349-42), APC-anti-human CD45 monoclonal antibody (Invitrogen, 17-0459-42), and APC-anti-human HLA-DR monoclonal antibody (Invitrogen, 12-9952-41) were used.

For intracellular protein detection, cells were stained with fluorophore-conjugated monoclonal antibodies according to established protocols. Briefly, Th1 or Th17 cells were stained with anti-mouse FITC-CD3 (Tonbo Biosciences, 35-0032) or anti-mouse PE-CD4 (Invitrogen, 11-0041-82) for 30 min at room temperature. For intercellular cytokine staining, the cells were fixed in 100 *μ*l of Fixation Buffer (Tonbo Biosciences, TNB-8222), vortexed at room temperature for 30 minutes, and then washed with Flow Staining Buffer. The cells were resuspended in permeabilization buffer (Tonbo Biosciences, TNB-1213) in the dark at room temperature for 5 minutes. Subsequently, the cells were stained with anti-mouse APC-IL17A (eBioscience, 17-7177-81) or anti-mouse APC-IFN-gamma (Tonbo Biosciences, 20-7311) for 30 min at 4°C. Finally, the cells were washed in Flow Staining Buffer. Cells were analyzed by flow cytometry using a FACSCalibur system (Becton Dickinson), and data were analyzed using FlowJo software.

### 2.5. IFN-*γ* and TNF-*α* Treatment hUC-MSCs

hUC-MSCs were seeded into 6-well plates (1 × 10^5^/well), and the medium was supplemented with combinations of the recombinant cytokines IFN-*γ* (PeproTech, AF-315-05) and TNF-*α* (PeproTech, AF-315-01A) (20 ng/ml). Cells were collected 24 h, 48 h, or 72 h after treatment.

### 2.6. CFSE Staining

CD3^+^ T cells were isolated from the spleen using immunomagnetic separation beads (Miltenyi Biotec, 130-095-130) according to the manufacturer's protocol. CD3^+^ T cells (10^6^/ml in PBS) were labeled with 5 *μ*M CFSE (Invitrogen, C34554) for 10 min at 37°C with gentle vortexing every 5 min. Labeling was terminated by adding a 5-fold volume of RPMI-1640 medium supplemented with 10% FBS.

### 2.7. T Cell Proliferation Assay

hUC-MSCs, sh-NC-MSCs, or sh-Chi3l1-MSCs (1 × 10^5^/well) were seeded into 24-well plates (2 × 10^6^/well) precoated with anti-CD3 antibody (2 *μ*g/ml) (BioLegend, 100309). After 6 h, CFSE-labeled CD3^+^ T cells (2 × 10^6^/well) were also seeded into 24-well plates and cocultured for another 3 days. T cell proliferation was analyzed by flow cytometry.

### 2.8. In Vitro Th17 Cell Differentiation

Naïve CD4 cells were isolated from C57BL/6J mouse spleens using immunomagnetic separation beads (Miltenyi Biotec, 130-106-643) according to the manufacturer's instructions and seeded into 96-well plates (5 × 10^5^/well) precoated with anti-CD3 antibody (5 *μ*g/ml) (BioLegend, 100309) with the addition of soluble anti-CD28 antibody (2 *μ*g/ml) (BioLegend, 102102). MSCs, sh-NC-MSCs, sh-Chi3l1-MSCs, or sh-Chi3l1-MSCs plus Stattic (20 *μ*M, Selleck, S7024) were seeded into 96-well plates (5 × 10^3^/well) 6 h before CD4 cell seeding. Th17 differentiation medium contained TGF-*β* (1.0 ng/ml) (PeproTech, AF-100-21C), IL-6 (30 ng/ml) (PeproTech, 216-16), IL-1*β* (20 ng/ml) (PeproTech, 211-11B), IL-23 (20 ng/ml) (BioLegend, 589002), anti-IL-4 (10 *μ*g/ml) (BioLegend, 504102), and anti-IFN-*γ* (10 *μ*g/ml) (BioLegend, 505833).

Th17 cells were differentiated for 72 h and restimulated with Cell Stimulation Cocktail (Tonbo Biosciences, TNB-4975) for 6 h before further analysis for intracellular cytokines by flow cytometry.

### 2.9. ELISA

The levels of IFN-*γ* and IL-17A in serum samples were determined using ELISA kits purchased from Invitrogen according to the manufacturer's instructions.

### 2.10. RNA Extraction, RT–PCR, and qPCR

Total RNA was extracted from the samples using TRIzol reagent (Invitrogen, 15596018). cDNA synthesis was conducted using a commercial reverse transcription kit (CWBIO, CW2020). Quantitative real-time PCR (qPCR) was performed using the UltraSYBR One-Step Kit (CWBIO, CW2624) on the 7500 Real-Time system analyzed using *ΔΔ*Ct calculations. GAPDH was used as the reference gene for normalization. Primer sequences are listed in Table 1.

### 2.11. Western Blot

Cells were lysed in RIPA buffer. The protein concentration of each sample was determined using a Protein Assay (Thermo Scientific, 23225). Protein samples (25 *μ*g) were loaded and separated on 10% SDS–PAGE gels and then transferred to polyvinylidene fluoride (PVDF) blotting membranes. PVDF membranes were blocked in TBST buffer containing 5% nonfat dry milk for 1 h. Rabbit anti-Chi3l1 (Cell Signaling, 47066S), rabbit anti-STAT3 (Cell Signaling, 12640), rabbit anti-p-STAT3 (Cell Signaling, 9145), and anti-GAPDH (Cell Signaling, 5174s) antibodies were incubated overnight at 4°C. Afterward, the mixture was incubated with HRP-conjugated secondary antibodies in blocking solution for 1 h at room temperature. Finally, enhanced chemiluminescence substrate (Thermo Scientific, 34578) was added to the membranes, and the proteins were assayed according to the manufacturer's instructions.

### 2.12. Mouse aGvHD Induction and Treatment

BALB/C mice (8 weeks old, female) were irradiated with a single dose of 800 cGy total body irradiation (TBI, Co^60^*γ* source). aGvHD mice were infused with bone marrow cells (1 × 10^7^) and splenocytes (1 × 10^7^) isolated from C57BL/6J mice (6 weeks old, male) through tail vein injection. Forty-eight hours after bone marrow transplantation, the recipient mice were administered hUC-MSCs, sh-Chi3l1-MSCs, sh-NC-MSCs (1 × 10^6^), or PBS (0.2 ml) via the tail vein.

### 2.13. H&E and Immunohistochemical Staining

Twenty-one days after treatment, the skin, liver, lung, and intestine from recipient mice were collected and fixed in 4% paraformaldehyde. The samples were then dehydrated by sequential treatment with 75% ethanol (1 h), 95% ethanol (1 h twice), and 95% ethanol (1 h twice). The samples were treated with xylene for 20 min twice before being embedded in paraffin. The samples were then sectioned at 5 *μ*m. Histology was performed using standard hematoxylin and eosin (H&E) staining. For immunohistochemical staining, the samples were sectioned at a thickness of 4 *μ*m and stained via dual-color immunohistochemical staining. Anti-IL-17A rabbit monoclonal primary antibody (Servicebio, GB11110) was used.

### 2.14. Statistical Analysis

Results are expressed as the mean ± SD. Unpaired Student's *t* test was performed to compare two mean values. One-way ANOVA and Tukey's multiple comparison tests were used to compare three or more mean values. The exact values of *n* and statistical significance are reported in the figures and the figure legends. Error bars represent the standard error of the mean (SEM). Significant differences in means are indicated as follows: ^∗^*P* < 0.05, ^∗∗^*P* < 0.01, and ^∗∗∗^*P* < 0.001.

## 3. Results

### 3.1. RNA-Seq Analyses of MSCs Obtained from Bone Marrow, Umbilical Cord, Amniotic Membrane, and Placenta Tissue

**Figure 1:**
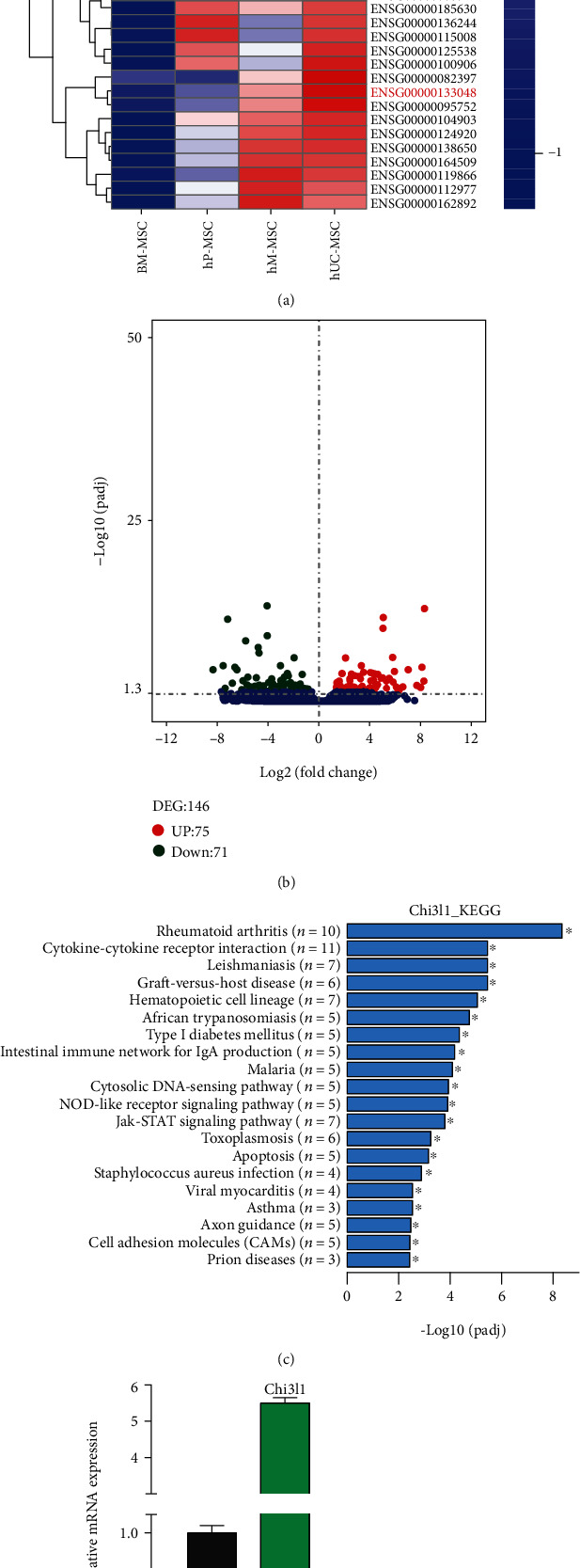
Chi3l1 is highly expressed in hUC-MSCs. (a) Heat map of differential gene expression in various MSCs, including BM-MSCs, hP-MSCs, hM-MSCs, and hUC-MSCs. (b) A volcano plot was used to analyze the DEGs (red indicates upregulated genes). (c) KEGG pathway indicating that Chi3l1 is highly associated with the JAK-STAT signaling pathway and graft-versus-host disease (GvHD) in hUC-MSCs. (d) Chi3l1 expression in different MSCs by qPCR. (e) hUC-MSCs were treated with IFN-*γ* and TNF-*α* (20 ng/ml) for 24 h, 48 h, and 72 h, and Chi3l1 mRNA levels were measured by qPCR. (f) The protein expression of Chi3l1 in IFN-*γ*- and TNF-*α*-pretreated hUC-MSC supernatants was analyzed by western blot. (g) The Chi3l1 protein expression levels in IFN-*γ*- and TNF-*α*-pretreated hUC-MSCs determined by western blot. JAK: Janus kinase; STAT: signal transducer and activator of transcription; IFN-*γ*: interferon-gamma; TNF-*α*: tumor necrosis factor-alpha.

Previous studies have identified several immunosuppressive molecules in MSCs from different tissues, including IDO, TSG6, and CD200 [1, 23]. To explore new molecules that affect MSC immunoregulatory capacity, we performed RNA-seq analysis of MSCs isolated from bone marrow, umbilical cord, amniotic membrane, and placenta tissues. We identified 146 differentially expressed genes (DEGs) that exhibited a more than 2-fold decrease or increase in mRNA expression (Figures 1(a) and 1(b) and Table S1). A heat map and volcano plot showing that Chi3l1 is highly expressed in hUC-MSCs are shown in Figures 1(a) and 1(b). In parallel, we performed a qPCR assay to compare mRNA expression across different MSCs. The results indicated that Chi3l1 was highly expressed in hUC-MSCs (Figure 1(d)). Gene set enrichment analysis showed that Chi3l1 was associated with JAK-STAT signaling, apoptosis, and graft-versus-host disease (Figure 1(c)). Chi3l1 has been implicated in inflammatory processes, including apoptosis, dendritic cell accumulation, and M2 macrophage differentiation. Moreover, previous studies revealed that treatment of macrophages with the proinflammatory cytokines IFN-*γ*, TNF-*α*, or IL-6 inflates Chi3l1 expression [24, 25]. Interestingly, we found that Chi3l1 expression was increased in IFN-*γ*- and TNF-*α*-pretreated hUC-MSCs and their supernatants, and a time-dependent relationship was observed (Figures 1(e)–1(g)). Several studies have indicated that treatment with the proinflammatory cytokines IFN-*γ* and TNF-*α* promotes immunosuppressive molecular expression in MSCs and thus could induce their immunosuppressive capacity [26, 27]. Together, we hypothesized that Chi3l1 may play a role in the immunosuppressive function of hUC-MSCs.

### 3.2. Knockdown of Chi3l1 Expression in hUC-MSCs

**Figure 2:**
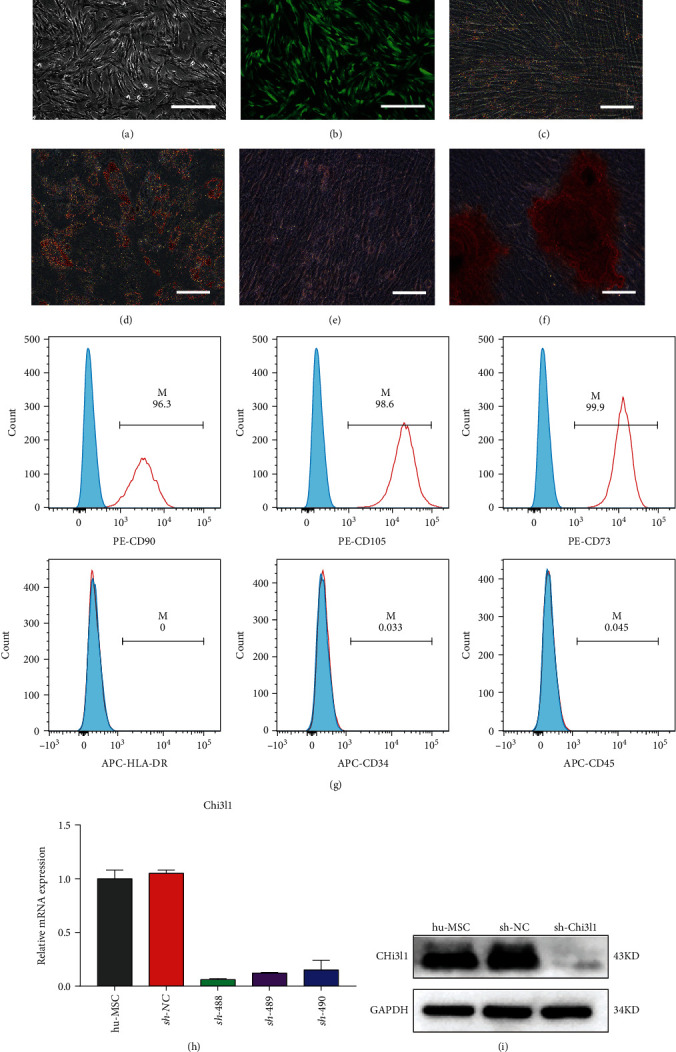
Chi3l1 knockdown in hUC-MSCs by Chi3l1-targeting shRNA lentiviral transfection. (a, b) The morphology of Chi3l1 knockdown hUC-MSCs (sh-Chi3l1-MSCs) shown by fluorescence microscopy. Scale bar: 400 *μ*m. (c, d) Oil red O staining was used to analyze the adipogenic ability of sh-Chi3l1-MSCs. (c) Shows the control group, and (d) shows the induced group. (e, f) Alizarin red staining was used to analyze the osteogenesis ability of sh-Chi3l1-MSCs. (e) Is the control group, and (f) is the induced group. Scale bar: 200 *μ*m. (g) Immunophenotypic analysis of sh-Chi3l1-MSCs was performed by flow cytometry. (h) mRNA expression levels of Chi3l1 in sh-Chi3l1-MSCs analyzed by qPCR. (i) Western blotting was used to assess Chi3l1 protein expression in sh-Chi3l1-MSCs. sh-NC: sh-NC-MSCs; sh-Chi3l1: sh-Chi3l1-MSCs.

To examine the effect of Chi3l1 on the immunomodulatory function of hUC-MSCs, hUC-MSCs were transfected with Chi3l1-targeting shRNA or control (NC) carried on a lentiviral vector. Transfection efficiency was examined by GFP expression via fluorescence microscopy analyses (Figure 2(b)). Reduced Chi3l1 expression in Chi3l1 knockdown hUC-MSCs (sh-Chi3l1-MSCs) was assessed by qPCR and western blot (Figures 2(h) and 2(i)). Next, the general characteristics of sh-Chi3l1-MSCs were investigated. The results showed that sh-Chi3l1-MSCs displayed a spindle-shaped appearance and adherent growth (Figures 2(a) and 2(b)) and expressed CD90, CD105, and CD106 (>95%) but not CD34, CD45, or human leukocyte antigen-DR (HLA-DR) (<2%) (Figure 2(g)) [28]. hUC-MSCs could also be differentiated into adipocytes and osteocytes (Figures 2(c)–2(f)). Moreover, hUC-MSCs transfected with control (NC) carried on lentiviral vector (sh-NC-MSCs) displayed similar general characteristics to sh-Chil3l1-MSCs (Figure S1). Taken together, these results demonstrated that Chi3l1 knockdown does not change the properties of hUC-MSCs.

### 3.3. Chi3l1 Deletion Impairs the Therapeutic Effects of hUC-MSCs in aGvHD Mice

**Figure 3:**
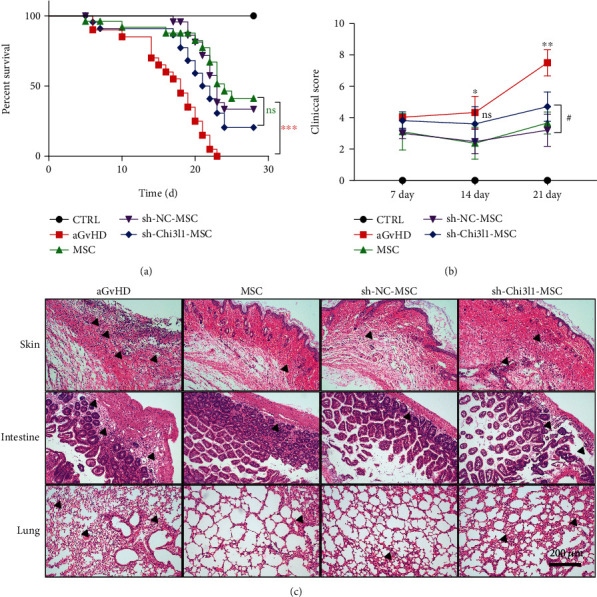
Chi3l1 deletion impairs the therapeutic effects of hUC-MSCs in aGvHD mice. (a–c) Recipient mice (BALB/C) were irradiated with a single dose of 800 cGy total body irradiation (Co^60^*γ* source) and intravenously injected with C57BL/6J bone marrow cells (1 × 10^7^) plus splenocytes (1 × 10^7^) to induce the aGvHD model. Two days after transplantation, different MSCs, including hUC-MSCs, sh-Chi3l1-MSCs, sh-NC-MSCs (1 × 10^6^), or PBS, were injected intravenously into aGvHD mice. (a) Survival curves of each group of mice (control: *n* = 12, aGvHD, hUC-MSCs, sh-Chi3l1-MSCs, and sh-NC-MSCs: *n* = 20, log-rank test, ^∗∗∗^*P* < 0.001). (b) The clinical score of hUC-MSCs, sh-Chi3l1-MSCs, sh-NC-MSCs, or PBS groups of mice. ^∗^*P* < 0.05; ^∗∗^*P* < 0.01; ns: not significant. (c) Hematoxylin and eosin staining was used to analyze the histological and pathological changes in the skin, intestine, and lung in aGvHD mice 21 days after hUC-MSCs, sh-Chi3l1-MSCs, sh-NC-MSCs, or PBS treatment. Scale bar: 200 *μ*m, *n* = 7, three independent experiments. CTRL: healthy mice.

To determine whether Chi3l1 is associated with hUC-MSC immunoregulation capacity in aGvHD, we established a mouse aGvHD model by transplantation of bone marrow cells and splenocytes obtained from C57BL/6J mice into recipient mice (BALB/C). HUC-MSCs, sh-Chi3l1-MSCs, sh-NC-MSCs, or PBS were intravenously injected into aGvHD mice 2 days after transplantation. We observed that PBS-treated mice displayed severe GvHD symptoms, including weight loss, reduced survival, and skin damage. Starting at 10 days, their survival time remarkably decreased, and the clinical score significantly increased. The other groups, which were hUC-MSC-, sh-NC-MSC-, and sh-Chi3l1-MSC-treated mice, exhibited different therapeutic effects (Figures 3(a) and 3(b)). Compared to the hUC-MSC and sh-NC-MSC groups, the survival time of sh-Chi3l1-MSC-treated mice was reduced, while the clinical score was enhanced starting on day 15 (Figures 3(a) and 3(b); *P* < 0.05, *P* < 0.01).

We next analyzed the histopathological lesions of the skin, intestine, and lung in hUC-MSC-, sh-Chi3l1-MSC-, sh-NC-MSC-, or PBS-treated mice. Compared to aGvHD treatment, hUC-MSC, sh-Chi3l1-MSC, and sh-NC-MSC treatment reduced lymphocyte infiltration and tissue injury in the skin, intestine, and lung, revealing that infusion into hUC-MSC, sh-Chi3l1-MSC, and sh-NC-MSC mice attenuated aGvHD mouse tissue injury (Figure 3(c)). Comparative analysis of the tissue injury in hUC-MSC-, sh-NC-MSC-, and sh-Chi3l1-MSC-treated mice revealed that the skin, intestine, and lung tissue in sh-Chi3l1-MSC-treated mice displayed increased lymphocyte infiltration and exacerbated tissue lesions (Figure 3(c)). These results illustrate that Chi3l1 deletion impairs the therapeutic benefits of hUC-MSCs in aGvHD mice.

### 3.4. Chi3l1 Deletion Reduces the Immunosuppressive Capacity of MSCs in aGvHD Mice

**Figure 4:**
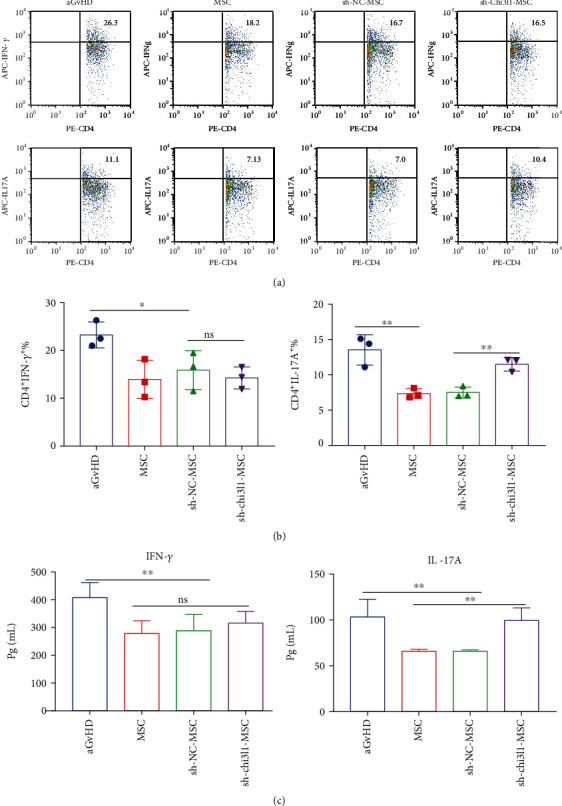
sh-Chi3l1-MSCs promote IL-17A production in aGvHD mice. (a, b) Lymphocytes were obtained from the spleens of aGvHD mice on day 14 after hUC-MSC, sh-Chi3l1-MSC, sh-NC-MSC (1 × 10^6^), or PBS (0.2 ml) treatment. Intracellular cytokines in lymphocytes were measured by flow cytometry. (a) IFN-*γ* and IL-17A were analyzed by flow cytometry. (b) The proportion of intracellular cytokines IFN-*γ* and IL-17A was measured in different groups. (c) Serum IFN-*γ* and IL-17A were measured by ELISA (*n* = 8, three independent experiments, one-way ANOVA, and Tukey's multiple comparison test, ^∗^*P* < 0.05; ^∗∗^*P* < 0.01; ns: not significant). IFN-*γ*: interferon-gamma; IL-17A: interleukine-17A.

To further validate Chi3l1-mediated hUC-MSC immunosuppression in aGvHD, we also investigated the changes in proinflammatory cytokines after hUC-MSC, sh-Chi3l1-MSC, sh-NC-MSC, or PBS treatment in mice. As expected, the percentages of CD4^+^ IFN-*γ* cells and CD4^+^IL-17A cells in the aGvHD group were significantly increased but were decreased in the hUC-MSC, sh-Chi3l1-MSC, and sh-NC-MSC groups (Figures 4(a) and 4(b); *P* < 0.05, *P* < 0.01). Additionally, the percentage of CD4^+^IL-17A cells was strikingly decreased in the hUC-MSC and sh-NC-MSC groups compared to the sh-Chi3l1-MSC group (Figures 4(a) and 4(b), *P* < 0.01), while CD4^+^ IFN-*γ* cells did not exhibit a significant difference (Figures 4(a) and 4(b)). Furthermore, we measured the concentrations of IL-17A and IFN-*γ* in plasma derived from hUC-MSC-, sh-Chi3l1-MSC-, sh-NC-MSC-, or PBS-treated mice using ELISA. Expression levels of IL-17A and IFN-*γ* were significantly increased in PBS-treated mice (Figure 4(c), *P* < 0.01). In the MSC treatment group, no significant differences in IFN-*γ* expression were observed in mice in the hUC-MSC, sh-Chi3l1-MSC, and sh-NC-MSC groups, but IL-17A expression levels in the sh-Chi3l1-MSC group were dramatically higher than those in the hUC-MSC and sh-NC-MSC groups (Figure 4(c), *P* < 0.01). Similar results for IL-17A and IFN-*γ* expression were found in the spleen. Consistent with RNA-seq analysis, these results demonstrate that Chi3l1 plays an important role in MSC-based aGvHD treatment.

### 3.5. hUC-MSC Secretion of Chi3l1 Relieves IL-17A Produced in the Intestine

**Figure 5:**
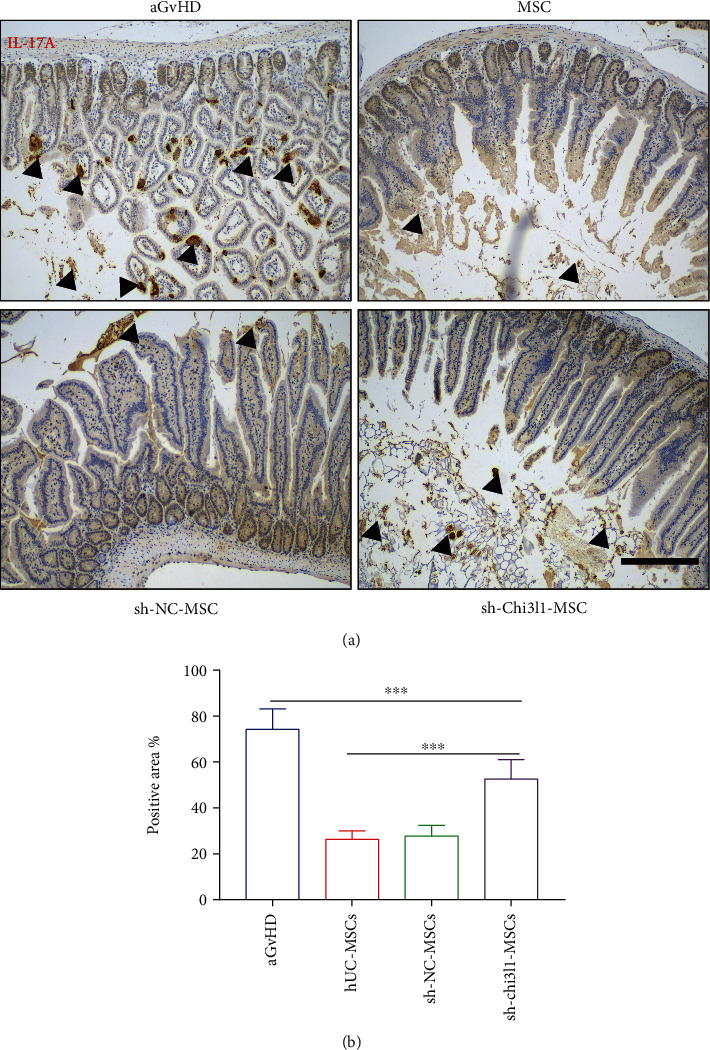
sh-Chi3l1-MSCs failed to repress IL-17A production in the intestine. (a) Intestinal immunohistochemical staining results of aGvHD mice on day 21 after hUC-MSC, sh-Chi3l1-MSC, sh-NC-MSC (1 × 10^6^), or PBS (0.2 ml) treatment. (b) Positive results of IL-17A expression measured using Image-Pro Plus software. Scale bar: 100 *μ*m, *n* = 7, three independent experiments, one-way ANOVA, and Tukey's multiple comparison test, *P* < 0.001.

IL-17A is a proinflammatory cytokine that is highly expressed during intestinal inflammation [29]. To further investigate the effects of Chi3l1 on hUC-MSCs attenuating IL-17A levels in the intestinal tissue, immunohistochemical staining was used to analyze the expression levels of IL-17A in the intestinal tissue from the various groups of mice. The results revealed increased IL-17A levels in aGvHD mouse intestines compared to hUC-MSC-, sh-Chi3l1-MSC-, and sh-NC-MSC-treated mice (Figures 5(a) and 5(b)). Comparative analysis of hUC-MSC-, sh-Chi3l1-MSC-, and sh-NC-MSC-treated mice revealed that IL-17A levels were higher in sh-Chi3l1-MSC mice (Figures 5(a) and 5(b)). These results demonstrated that sh-Chi3l1-MSCs fail to inhibit IL-17A production in the intestine of aGvHD mice. Together with the results shown in Figure 4, these findings demonstrate that Chi3l1 is necessary for hUC-MSC regulation of Th17 differentiation.

### 3.6. hUC-MSCs with Chi3l1 Knockdown Exhibit Reduced Ability to Repress T Cell Proliferation

**Figure 6:**
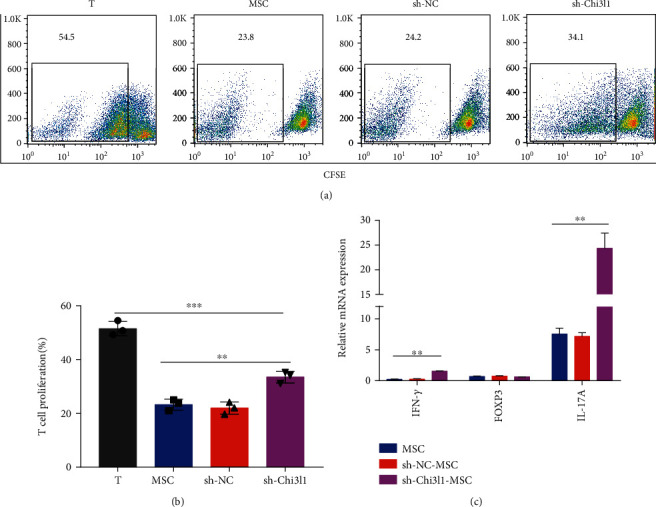
Chi3l1 deletion compromises hUC-MSCs and inhibits T cell proliferation. (a–c) CD3^+^ T cells obtained from the spleens of healthy C57BL/6J mice were stained with CFSE (5 *μ*M) and then incubated in 24-well plates with hUC-MSCs, sh-Chi3l1-MSCs, and sh-NC-MSCs at a CD3^+^ T/hUC-MSC ratio of 20 : 1 for 72 hours. (a) Proliferation of CD3^+^ T cells analyzed by flow cytometry. (b) CD3^+^ T cell proliferation was measured after treatment with different hUC-MSCs. (c) mRNA expression levels of IFN-*γ*, IL-17A, and FOXP3 analyzed by qPCR (three independent experiments, one-way ANOVA, and Tukey's multiple comparison test, ^∗∗^*P* < 0.01; ns: not significant).

Previous studies have shown that hUC-MSCs, which repress the proinflammatory response, inhibit T cell proliferation and differentiation into Th1 or Th17 cells [5, 30]. As shown in Figure 6, hUC-MSCs and sh-NC-MSCs both inhibited T cell proliferation, while the capacity of sh-Chi3l1-MSCs to inhibit T cell proliferation was decreased (Figures 6(a) and 6(b), *P* < 0.01). Notably, our finding is compatible with recent study results, which also indicated that Chi3l1 is associated with the ability of hUC-MSCs to inhibit T cell proliferation [20]. We next analyzed IFN-*γ*, IL-17A, and Foxp3 mRNA levels in cocultured T cells. IL-17A and IFN-*γ* expression in sh-Chi3l1-MSCs was increased compared to that in hUC-MSCs and sh-NC-MSCs (Figure 6(c), *P* < 0.01). These results suggest that Chi3l1 is involved in the process by which hUC-MSCs regulate T cell proliferation.

### 3.7. hUC-MSC-Secreted Chi3l1 Represses CD4 T Cell Differentiation to Th17 Cells by Inhibiting STAT3 Activation

**Figure 7:**
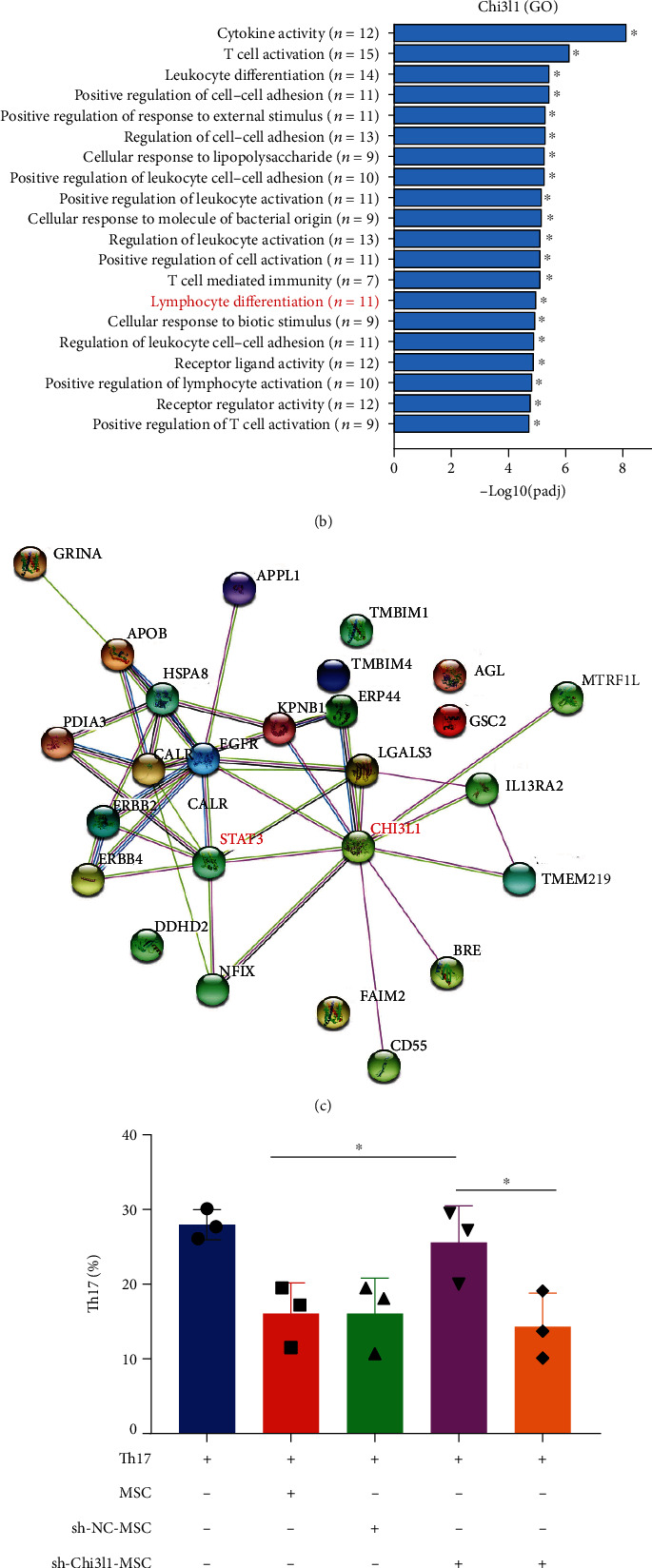
hUC-MSC-secreted Chi3l1 represses CD4 differentiation to Th17 cells by inhibiting STAT3 activation. (a) Reactome pathway showing that Chi3l1 is associated with interleukin-6 family signaling. (b) Gene Ontology showing that Chi3l1 is associated with lymphocyte differentiation. (c) STRING interaction network displaying that Chi3l1 interacts with STAT3. (d–f) CD4^+^ T cells (5 × 10^5^) obtained from the spleen of C57BL/6J mice were differentiated into Th17 cells in 96-well plates in the presence or absence of hUC-MSCs, sh-NC-MSCs, sh-Chi3l1-MSCs, or sh-Chi3l1-MSCs (5 × 10^3^) plus Stattic (20 *μ*M) at a CD4^+^ T/hUC-MSC ratio of 100 : 1 for 72 hours. (e) The proportion of Th17 cells was analyzed using flow cytometry. (d) The percentage of Th17-positive cells was detected by flow cytometry (one-way ANOVA and Tukey's multiple comparison test, ^∗^*P* < 0.05, three independent experiments). (f) Protein expression level of p-STAT3 measured by western blot. STAT3: signal transducer and activator of transcription 3; sh-NC: sh-NC-MSCs; sh-Chi3l1: sh-Chi3l1-MSCs. Stattic: p-STAT3 inhibitor.

**Figure 8:**
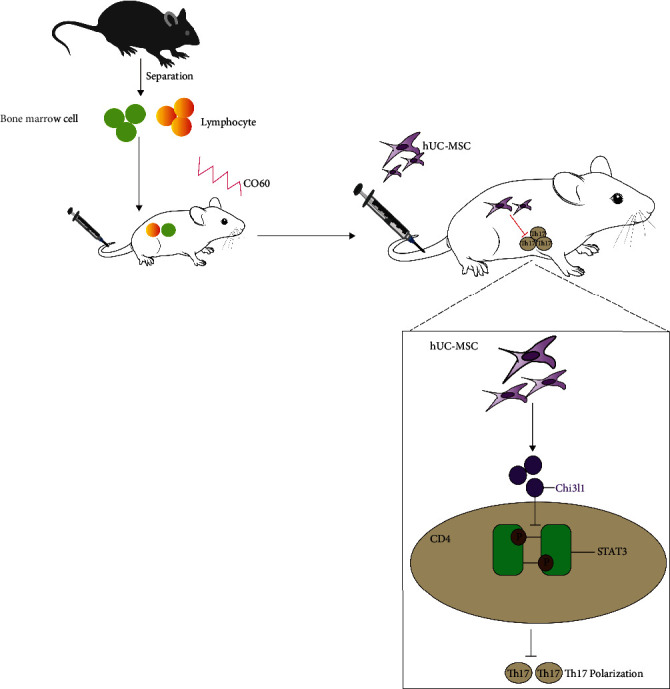
Model of the function and mechanism by which hUC-MSC-secreted Chi3l1 regulates CD4 differentiation into Th17 cells.

To investigate whether hUC-MSC-secreted Chi3l1 is linked to Th17 differentiation, we analyzed Chi3l1 function in an RNA-seq dataset. The Reactome pathway results indicated that Chi3l1 was implicated in the IL-6 signaling pathway and lymphocyte differentiation activity (Figures 7(a) and 7(b)). In addition, STRING database analysis revealed that Chi3l1 interacts with STAT3 (Figure 7(c)). Since the IL-6 signaling pathway and STAT3 activation are strongly associated with Th17 differentiation [31], we performed Th17 cell differentiation assays. Compared to hUC-MSCs and sh-NC-MSCs, the proportion of Th17 cells was increased in sh-Chi3l1-MSCs (Figures 7(d) and 7(e), *P* < 0.05). These results demonstrate that hUC-MSC-secreted Chi3l1 is associated with the process by which hUC-MSCs inhibit Th17 differentiation. Furthermore, we used Stattic, a p-STAT3 inhibitor, to block STAT3 activation during CD4 T cell differentiation to Th17 cells. Flow cytometry results revealed that blocking STAT3 activation reduced the proportion of Th17 cells in the sh-Chi3l1-MSC group (Figures 7(d) and 7(e), *P* < 0.05). We further examined p-STAT3protein levels in CD4 cells. The results showed that p-STAT3 levels were remarkably increased in CD4 cells in the presence of sh-Chi3l1-MSCs, while the addition of Stattic led to a decrease in p-STAT3 levels (Figure 7(f)). Taken together, our results demonstrate that hUC-MSC-secreted Chi3l1 represses Th17 differentiation by inhibiting STAT3 activation (Figure 8).

## 4. Discussion

Multiple clinical studies have confirmed the safety of both allogeneic and autologous MSCs for the treatment of aGvHD [11, 32]. hUC-MSC-based therapies for aGvHD have made significant advances in recent years [33]. Although a series of factors are known to be critical for hUC-MSC immunoregulation, but the complete immunomodulatory mechanism of hUC-MSCs in aGvHD treatment is still unclear. The present study provides the first report on alterations in the immunomodulatory functions of hUC-MSCs induced by Chi3l1.

Chi3l1 is secreted by activated macrophages, chondrocytes, neutrophils, and synovial cells [34]. Previous studies have determined that Chi3l1 plays a role in the Th2 inflammatory response and IL-13-induced inflammation [35, 36], regulating allergen sensitization, apoptosis, dendritic cell accumulation, and M2 macrophage differentiation [37, 38]. In this study, we found that Chi3l1 mRNA was more highly expressed in hUC-MSCs than in bone marrow, amniotic membrane, and placenta-derived MSCs and exhibited a remarkable association with GvHD and inflammation. The proinflammatory cytokine IFN-*γ* combined with TNF-*α*- or IL-1*β*-pretreated MSCs was demonstrated to enhance anti-inflammatory molecules or exosomes and “license” MSC immunoregulatory ability [26, 39, 40]. Unexpectedly, our finding was similar to these results, in which Chi3l1 expression was associated with hUC-MSCs exposed to IFN-*γ* and TNF-*α* in vitro. Combined with the RNA-seq data, we hypothesized that Chi3l1 may be a potential immunoregulatory molecule of hUC-MSCs for the treatment of aGvHD.

In our study, using Gene Ontology and KEGG database analysis, we found that Chi3l1 was engaged in T cell proliferation and differentiation and the aGvHD process. Next, stable transfection of hUC-MSCs was performed using Chi3l1 knockdown lentiviral vectors. The results of in vitro T cell proliferation analysis indicated that sh-Chi3l1-MSC restrained the suppression of T cell proliferation and IL-17A expression. This study in an aGvHD mouse model revealed that Chi3l1 knockdown impaired the therapeutic efficacy of hUC-MSCs, manifesting as reduced survival time, higher clinical scores, and increased lymphocyte infiltration in aGvHD organs, including the skin, intestine, and lung. In addition, sh-Chi3l1-MSC transplantation significantly increased the proportion of Th17 cells in aGvHD mice and upregulated expression of IL-17A. Together, these data suggest that Chi3l1 plays an important role in hUC-MSC immunosuppression capacity and is implicated in hUC-MSC therapeutic potential in aGvHD.

Of note, the immunoregulatory factors secreted by MSCs and their cell signaling pathways may influence the immunosuppressive capacity and therapeutic effects of MSCs. In our study, the gene set enrichment analysis results indicated that Chi3l1 may play a role in the IL-6-STAT3 pathway and be associated with lymphocyte differentiation. Previous studies demonstrated that STAT3 activation is a crucial component of IL-6-mediated regulation of Th17 cell differentiation [41]. IL-17A has been implicated in many inflammatory diseases, such as rheumatoid arthritis, asthma, systemic lupus erythematosus (SLE), and allograft rejection [42]. Our in vitro study found that the proportion of Th17 cells was increased and p-STAT3 was more activated in the sh-Chi3l1-MSC group than in the hUC-MSC group and sh-NC-MSC group, but the addition of a p-STAT3 inhibitor rescued this phenomenon. In addition, sh-Chi3l1-MSCs infusion in aGvHD mice impaired the ability to inhibit IL-17A production in the spleen, serum, and intestine. Taken together, we revealed that Chi3l1 is engaged in hUC-MSCs restraining Th17 cell polarization via p-STAT3 expression, but it remains unclear precisely how Chi3l1 interacts with STAT3 activation.

Until to now, MSC inhibition Th17 cell differentiation is well known, and the complete mechanism is still unclear [43]. In our study, we uncovered that the secreted protein Chi3l1 was highly expressed in hUC-MSCs, and it was associated with CD4 differentiation into Th17 cells through repression STAT3 activation. In vivo experimental results demonstrated that Chi3l1 plays an important role in hUC-MSC therapeutic benefits in aGvHD by inhibiting Th17 cell differentiation to reduce the inflammatory response. Overall, our findings demonstrated that Chi3l1 is a novel immunosuppressive molecule that is associated with hUC-MSCs regulating Th17 differentiation and attenuating aGvHD symptoms (Figure 8). These results may aid in the development of cell-based therapies for the treatment of aGvHD.

## 5. Conclusions

The present study reveals that a new immunosuppressive molecule Chi3l1 regulates the function of hUC-MSCs. Although our study uncovers that hUC-MSC-secreted Chi3l1 could regulate Th17 differentiation by inhibiting STAT3 activation and attenuate the aGvHD symptoms, but the complete mechanism of Chi3l1 inhibition STAT3 activation still needs further investigation. However, these innovative insights into the mechanisms through which hUC-MSCs regulate immune responses may improve the clinical utility of these cells in aGvHD by targeting Chi3l1, which may provide a novel accessible strategy to improve the therapy effect of hUC-MSCs for aGvHD.

## Figures and Tables

**Table 1:** Primer sequences.

Gene	Primer sequence (5′–3′)
Chi3l1 forward	TACGGCATGCTCAACACACT
Chi3l1 reverse	TGCCCATCACCAGCTTACTG
GAPDH forward	TCAAGATCATCAGCAATGCC
GAPDH reverse	CGATACCAAAGTTGTCATGGA
IL-17A forward	TTCATCTGTGTCTCTGATGC
IL-17A reverse	GAGCTTTGAGGGATGATCG
Foxp3 forward	TCCTTCCCAGAGTTCTTCC
Foxp3 reverse	GATAAGGGTGGCATAGGTG
IFN-*γ* forward	CACCTGATTACTACCTTCTTCAG
IFN-*γ* reverse	GTTGTTGACCTCAAACTTGG

## Data Availability

The datasets used and/or analyzed during the current study are available from the corresponding author on reasonable request.

## Abbreviations

HUC-MSCs: Human umbilical cord mesenchymal stem cells
Chi3l1: Chitinase-3-like protein 1
aGvHD: Acute graft-versus-host disease
IFN-*γ*: Interferon-gamma
TNF-*α*: Tumor necrosis factor-alpha
Th17: T-helper 17 cell
STAT3: Signal transducer and activator of transcription 3
IL-17A: Interleukin-17A.

## Supplementary Materials

Supplementary 1 Table S1: the DEGs of four MSCs.

Supplementary 2 Figure S1: control (NC) lentivirus transfection of hUC-MSCs.
