# Supplementary material for: hUC-MSCs Attenuate Acute Graft-Versus-Host Disease through Chi3l1 Repression of Th17 Differentiation
Source: Stem Cells Int. 2022 Oct 12;2022:1052166. doi: 10.1155/2022/1052166 (PMC9582900; doi:10.1155/2022/1052166)
Supplement: Supplementary 1 — Table S1: the DEGs of four MSCs. [file 1052166.f1.pdf]

geneid	M	U	B	P
ENSG0000	637.9091	806.6885	587.4434	266.9478
ENSG0000	0.98243	0.048574	4.0113	0.912038
ENSG0000	0.024602	2.241413	0.041762	0.008185
ENSG0000	0.44379	7.844517	6.237132	0.269949
ENSG0000	0.521614	0.022564	0.449184	3.784632
ENSG0000	4.12728	7.782162	8.645267	0.271908
ENSG0000	0.382284	0.040218	29.30471	0.773726
ENSG0000	1.49379	0.044693	2.486535	2.722585
ENSG0000	2.855235	0.360433	0.0266	11.40366
ENSG0000	2.737668	0.266969	3.700502	8.070899
ENSG0000	1.597176	2.646509	3.239013	0.708156
ENSG0000	43.59145	4.417988	28.84989	19.60345
ENSG0000	0.086897	1.42707	0.222762	0.028844
ENSG0000	3.182227	0.60034	6.400256	5.590439
ENSG0000	3.654327	2.028306	0.382428	0.228241
ENSG0000	0.658667	2.132983	0.370139	0.049899
ENSG0000	0.839795	0.989504	0.488042	0.046405
ENSG0000	0.172176	0.437368	0.020579	0.008378
ENSG0000	21.50319	69.06212	2.718341	6.938861
ENSG0000	3.556456	19.22239	5.478543	1.325247
ENSG0000	12.52755	1.685614	0.452353	10.83598
ENSG0000	28.86772	67.90498	8.753271	21.93478
ENSG0000	140.3405	172.0238	78.48527	56.14945
ENSG0000	1.556393	2.132367	1.379546	0.415213
ENSG0000	1.322367	0.96156	1.738383	2.744486
ENSG0000	1.139772	1.884865	0.696211	0.1058
ENSG0000	0.116523	0.462635	0.034697	0.073608
ENSG0000	0.323574	1.347607	1.375524	0.079664
ENSG0000	1.601221	0.009265	0.180173	3.831646
ENSG0000	0.268	0.089744	0.065872	0.676252
ENSG0000	9.624184	1.869933	9.650911	11.37282
ENSG0000	43.32326	55.56486	7.268645	7.219056
ENSG0000	0.375388	1.700188	0.130583	0.014576
ENSG0000	0.120898	0.732277	0.517041	0.052051
ENSG0000	0.233793	0.698422	0.005751	0.083005
ENSG0000	4.952158	14.67259	0.510156	3.112738
ENSG0000	0.022156	1.061767	0.040455	0.061978
ENSG0000	16.10586	1.53655	2.109788	23.24918
ENSG0000	1.016305	0.159628	0.361956	1.461436
ENSG0000	0.178873	0.001957	1.992355	0.325525
ENSG0000	1.892989	1.526877	2.467539	0.322537
ENSG0000	2.476231	0.108894	0.238336	3.788999
ENSG0000	0.388471	3.839364	8.231182	0.136198
ENSG0000	0.004764	0.10365	0.024321	0.005025
ENSG0000	7.439021	9.529159	11.90864	3.594767
ENSG0000	0.08573	0.024065	1.447994	0.423654
ENSG0000	0.004515	0.180875	0.002813	0.003858
ENSG0000	30.3475	47.60219	64.83922	20.40497
ENSG0000	2.129482	0.807173	3.855957	4.353785
ENSG0000	5.024736	0.266016	0.482819	14.6955
ENSG0000	0.048189	14.36602	0	0.061359
ENSG0000	0.794284	0.170801	0.840865	1.211734
ENSG0000	0.416369	0.162911	0.638999	0.989609
ENSG0000	4.927102	1.50882	6.610707	4.801475
ENSG0000	1.00579	0.023884	0.033383	5.210642
ENSG0000	0.703524	0.263542	0.2315	2.521628
ENSG0000	0.001855	0.081375	0.517278	0

ENSG0000	1.205972	0.205674	0.301157	2.887563
ENSG0000	0.064252	0.005069	0.036327	0.113539
ENSG0000	3.066824	1.038744	0.398409	4.417124
ENSG0000	0.066576	0.35098	0.030846	0.037918
ENSG0000	0.692262	5.981668	0.087668	0.173799
ENSG0000	0.022072	0.244558	0.005324	0
ENSG0000	0.955054	0.94807	0.071934	0.184716
ENSG0000	2.91411	7.786633	1.0895	2.767253
ENSG0000	0.571279	0.223227	0.003354	2.969957
ENSG0000	0.039667	0.1681	0.697974	0.038675
ENSG0000	32.23923	4.193465	9.548465	36.96387
ENSG0000	0.22547	1.081806	0.177469	0.176102
ENSG0000	4.985683	11.5384	3.013069	2.8172
ENSG0000	93.47537	108.3542	209.365	5.025077
ENSG0000	0.700882	1.491256	0.88699	0.1657
ENSG0000	6.431283	6.577985	2.818747	2.91158
ENSG0000	0.103479	0.04311	0.253597	1.013533
ENSG0000	5.670619	8.412554	20.43041	3.574049
ENSG0000	0.040631	0.446793	0.137955	0.050528
ENSG0000	1.307926	7.079118	2.963523	2.592785
ENSG0000	0.157212	0.671367	0.041542	0.03718
ENSG0000	1.209357	0.624746	1.074992	2.453886
ENSG0000	0.010829	0.11715	0.087996	0.008033
ENSG0000	1.995905	0.086512	6.691321	2.541177
ENSG0000	4.459677	0.417017	0.643977	4.543878
ENSG0000	6.514776	4.046697	3.765685	10.19422
ENSG0000	1.380489	1.101511	0.212028	0.228003
ENSG0000	0.058032	0.678803	0.004158	0.049339
ENSG0000	0.618451	0.044142	0.062438	1.692519
ENSG0000	2.380668	1.401987	2.180429	2.914015
ENSG0000	0.100125	0	0.002836	0.134915
ENSG0000	0.727298	0.019382	0.039289	0.969008
ENSG0000	0.075084	2.298775	0.301021	0.139012
ENSG0000	1.667722	1.102376	2.764394	2.502394
ENSG0000	0.021223	0.009823	0.19719	0.214035
ENSG0000	35.24502	41.31088	231.9718	15.63122
ENSG0000	1.732927	0.57995	0.57833	3.040074
ENSG0000	0.752208	0.200004	7.838759	3.046364
ENSG0000	0.041457	0.1322	0.53869	0.010898
ENSG0000	3.509159	7.361781	1.349686	1.447297
ENSG0000	6.183881	7.419571	11.6399	0.387219
ENSG0000	0.114813	1.01705	0	0.069252
ENSG0000	4.671345	6.481277	3.872589	2.720303
ENSG0000	0.12395	0.008002	0.55043	0.132261
ENSG0000	0.267148	1.222637	0.366904	0.098556
ENSG0000	3.189365	1.9947	1.817159	0.250173
ENSG0000	11.40094	0.971799	0.994927	9.691507
ENSG0000	0.374976	0.035572	0.14995	0.85219
ENSG0000	0.063396	0.556846	0	0
ENSG0000	0.04893	0.001672	0.001803	0.161511
ENSG0000	1.708977	1.116365	0.88981	3.076447
ENSG0000	1.691331	3.425912	2.364332	0.392326
ENSG0000	0.660948	0.259779	0.801904	1.063953
ENSG0000	0.354882	0.206411	0.890899	0.776195
ENSG0000	0.094521	0.398709	0.585892	0.065882
ENSG0000	2.719152	0.477895	0.049175	34.63235
ENSG0000	8.534776	1.840338	1.595569	14.99652
ENSG0000	19.22864	7.303262	0.115361	53.79641

ENSG0000	0.13815	1.330116	0.732796	0.273338
ENSG0000	1.53194	0.148103	0.862849	1.680763
ENSG0000	0.482019	0.286526	1.502845	2.334839
ENSG0000	12.40842	11.76496	31.26504	3.145915
