# Supplementary material for: hUC-MSCs Attenuate Acute Graft-Versus-Host Disease through Chi3l1 Repression of Th17 Differentiation
Source: Stem Cells Int. 2022 Oct 12;2022:1052166. doi: 10.1155/2022/1052166 (PMC9582900; doi:10.1155/2022/1052166)
Supplement: Supplementary 2 — Figure S1: control (NC) lentivirus transfection of hUC-MSCs. [file 1052166.f2.pdf]

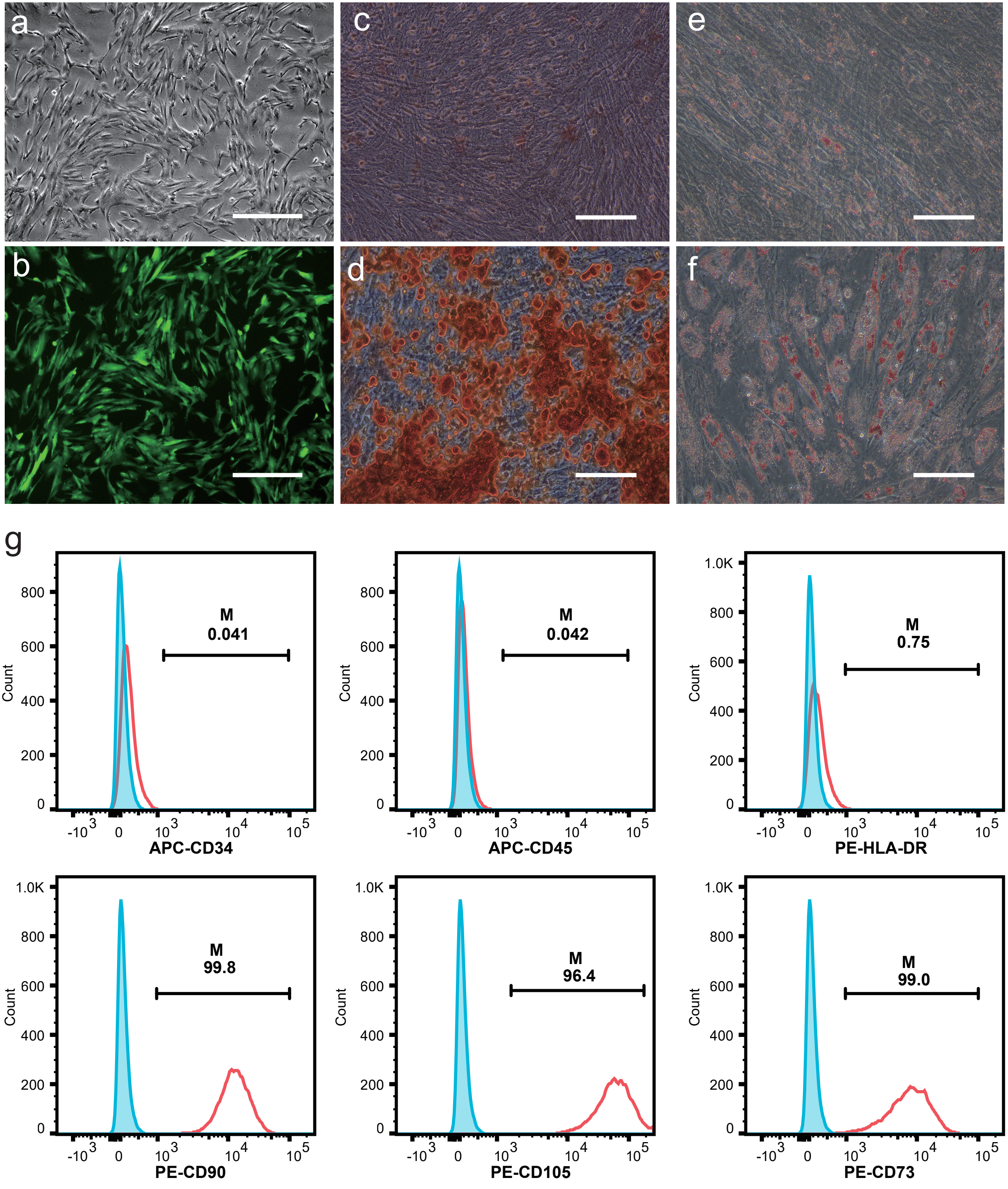
a
b
c
d
e
f
g
M 0.041
Count
APC-CD34
M 0.042
APC-CD45
M 0.75
PE-HLA-DR
M 99.8
PE-CD90
M 96.4
PE-CD105
M 99.0
PE-CD73
